# Development of an automated artificial intelligence-based tool for reticulin fibrosis assessment in bone marrow biopsies

**DOI:** 10.1007/s00428-025-04122-5

**Published:** 2025-05-13

**Authors:** Giuseppe D’Abbronzo, Antonio D’Antonio, Annarosaria De Chiara, Luigi Panico, Lucianna Sparano, Anna Diluvio, Antonello Sica, Gino Svanera, Giovanni De Chiara, Mariano Fuggi, Ferdinando Russo, Renato Franco, Andrea Ronchi

**Affiliations:** 1 Pathology Unit, Department of Mental and Physical Health and Preventive Medicine, Università Degli Studi Della Campania “Luigi Vanvitelli”, Via Luciano Armanni 5, 80138 Naples, Italy; 2 Pathology Unit, Hospital “Ospedale del Mare”, 80147 Naples, Italy; 3 Histopathology of Lymphomas and Sarcoma SSD, Istituto Nazionale Dei Tumori I.R.C.C.S. Fondazione “Pascale”, 80131 Naples, Italy; 4 Pathology Unit, Hospital “Monaldi”, 80131 Naples, Italy; 5 Pathology Unit, Hospital “Andrea Tortora”, 82100 Pagani, Italy; 6 Haematology and Oncology Unit, Vanvitelli Hospital, 80131 Naples, Italy; 7 Haematology Unit, ASL Na2 North, 80014 Giugliano, Italy; 8 Pathology Unit, Hospital “A.O.R.N. San Giuseppe Moscati”, 83100 Avellino, Italy; 9 Vanvitelli Hospital, 80138 Naples, Italy

**Keywords:** Myeloproliferative neoplasms, Reticulin fibrosis, Primary myelofibrosis

## Abstract

Bone marrow fibrosis plays a critical role in the diagnosis, prognosis, and management of haematological disorders, particularly myeloproliferative neoplasms like primary myelofibrosis. Accurate assessment of fibrosis, typically graded through histochemical techniques such as reticulin and trichrome staining, is essential but remains highly dependent on the pathologist’s experience. To address the challenges of variability in interpretation and the increasing demand for standardized evaluations, we developed a digital pathology system for automated bone marrow reticulin fibrosis grading. This study utilized 86 bone marrow biopsy specimens from patients diagnosed with Philadelphia chromosome-negative myeloproliferative neoplasms, collected between 2018 and 2023. A fully convolutional network based on the InceptionV3 architecture was trained to assess fibrosis grades (MF0–MF3) from whole slide images of reticulin-stained sections. The model was trained using 3814 annotated images and validated using a separate set of 40 BMBs. The algorithm’s performance was evaluated by comparing its fibrosis grading to expert hematopathologists’ assessments, yielding a Cohen’s kappa coefficient of 0.831, indicating excellent agreement. The algorithm showed strong concordance in fibrosis grading, especially for MF0 (*k* = 0.918) and MF3 (*k* = 0.886), and substantial agreement for intermediate grades (MF1 and MF2). Further validation across multiple institutions and scanning platforms confirmed the algorithm’s robustness, with an overall agreement of 0.816. These results demonstrate the potential of digital pathology tools to provide standardized, reproducible fibrosis grading, thereby aiding pathologists in clinical decision-making and training.

## Introduction

Evaluating fibrosis in a bone marrow biopsy (BMB) is critical for diagnosing, staging, and managing various haematological and systemic disorders. The assessment of bone marrow fibrosis typically involves analyzing the degree of reticulin or collagen fibre deposition in the marrow stroma, graded using a standardized scale (e.g., WHO grading system) [1]. The evaluation has several key roles: diagnosis of myeloproliferative neoplasms (MPNs), defining prognosis, monitoring disease progression, therapeutic guidance [2–4].

Bone marrow fibrosis is a hallmark feature of primary myelofibrosis (PMF), a type of MPN. Its presence, pattern, and severity help differentiate PMF from other MPNs like polycythemia vera (PV) or essential thrombocythemia (ET) [5]. Fibrosis can also be secondary to progression from other MPNs to a more advanced fibrotic stage. The degree of fibrosis correlates with disease severity and prognosis: advanced fibrosis (e.g., MF-3) often indicates a worse prognosis and higher risk of complications such as cytopenias and extramedullary haematopoiesis [6].

Sequential biopsies to evaluate fibrosis can track disease evolution or response to therapy, particularly in patients with MPNs, acute leukaemia, or myelodysplastic syndromes (MDS) with fibrosis [7].

Worsening fibrosis might signal disease progression or therapy resistance. Fibrosis grading may influence treatment decisions, such as considering allogeneic stem cell transplantation in advanced myelofibrosis, and is important for assessing the efficacy of antifibrotic treatments like JAK inhibitors [8]. Fibrosis may occur in various conditions beyond primary hematologic diseases, including infections (e.g., tuberculosis, fungal infections), metastatic cancers involving the bone marrow, autoimmune disorders (e.g., systemic lupus erythematosus), and chronic inflammatory states (e.g., sarcoidosis) [3].

Assessment of bone marrow fibrosis mainly relies on histological evaluations and involves histochemical techniques such as reticulin stain, which highlights reticulin fibres, and trichrome stain, which identifies collagen fibres, indicating advanced fibrosis [1].

Despite the fundamental importance of such an evaluation, it remains operator-dependent, and therefore, its accuracy relies on the pathologist’s experience. The reproducibility of reticulin fibrosis grading has been evaluated by various research groups, employing either a five-tier scoring system or the WHO-endorsed classification. Reported concordance rates have reached as high as 88% [9–14].

Therefore, a bone marrow reticulin fibrosis assessment system that is reproducible, operator-independent, and capable of assisting pathologists during their training would be of great clinical utility.

In this setting, digital pathology is developing worldwide as a system that can support standardization in anatomical pathology [15].

In a previous study, we developed a digital system for assessing bone marrow cellularity [16].

The aim of this study was to develop a digital system for evaluating bone marrow reticulin fibrosis.

## Materials and methods

### Case selection and digital image scanning

BMB specimens from the archives of the Pathology Department at the University Hospital “Luigi Vanvitelli” (Italy) were retrospectively selected for analysis. The cases, collected between January 2018 and June 2023, were chosen based on the following inclusion criteria:

- Patient age ≥ 18 years
- Biopsy fragment length ≥ 1 cm
- Availability of complete clinical and histological data
- Diagnosis of Philadelphia chromosome-negative myeloproliferative neoplasms (Ph-MPNs)
- Fibrosis grading documented in accordance with the World Health Organization (WHO) guidelines [1]

A total of 86 BMB specimens met these criteria and were subsequently reviewed by an expert hematopathologist. In all the cases, there was agreement between the data in the archive and the re-evaluation performed by the expert hematopathologist regarding both the diagnosis of Ph-MPN and the degree of fibrosis. Finally, 50 cases were deemed appropriate for further analysis and digital scanning.

For each selected case, a histological slide stained for reticulin was prepared. The slide was stained by Reticulum II Staining Kit (Ventana Medical Systems, Inc. 1910 E. Innovation Park Drive Tucson, Arizona 85755 USA) using an automatic staining system (BenchMark Special Stains system, Ventana Medical Systems, Inc. 1910 E. Innovation Park Drive Tucson, Arizona 85755 USA). This stain is a modification of Gordon and Sweets Stain, is commonly used for the assessment of fibrosis in bone marrow biopsies, and highlights reticular fibres. All the slides were scanned using the Ventana DP200 Slide Scanner (Roche Diagnostics S.p.A., Monza, Italy). The resulting whole slide images (WSIs) were exported for analysis. The scanned slides were then randomly allocated into two sets: a training set comprising 10 slides and a validation set comprising 40 slides. In particular, the validation set included 8 cases diagnosed as MF-0, 9 cases diagnosed as MF-1, 11 cases diagnosed as MF-2, and 12 cases diagnosed as MF-3.

### Training set production and tool development

An expert hematopathologist and a properly trained resident annotated the hematopoietic tissue in the 10 slides designated for the training set. They assessed the degree of reticulin fibrosis for each annotation according to the recommendations of the WHO [1] using the software QuPath V 4.3 [17].

**Fig. 1:**
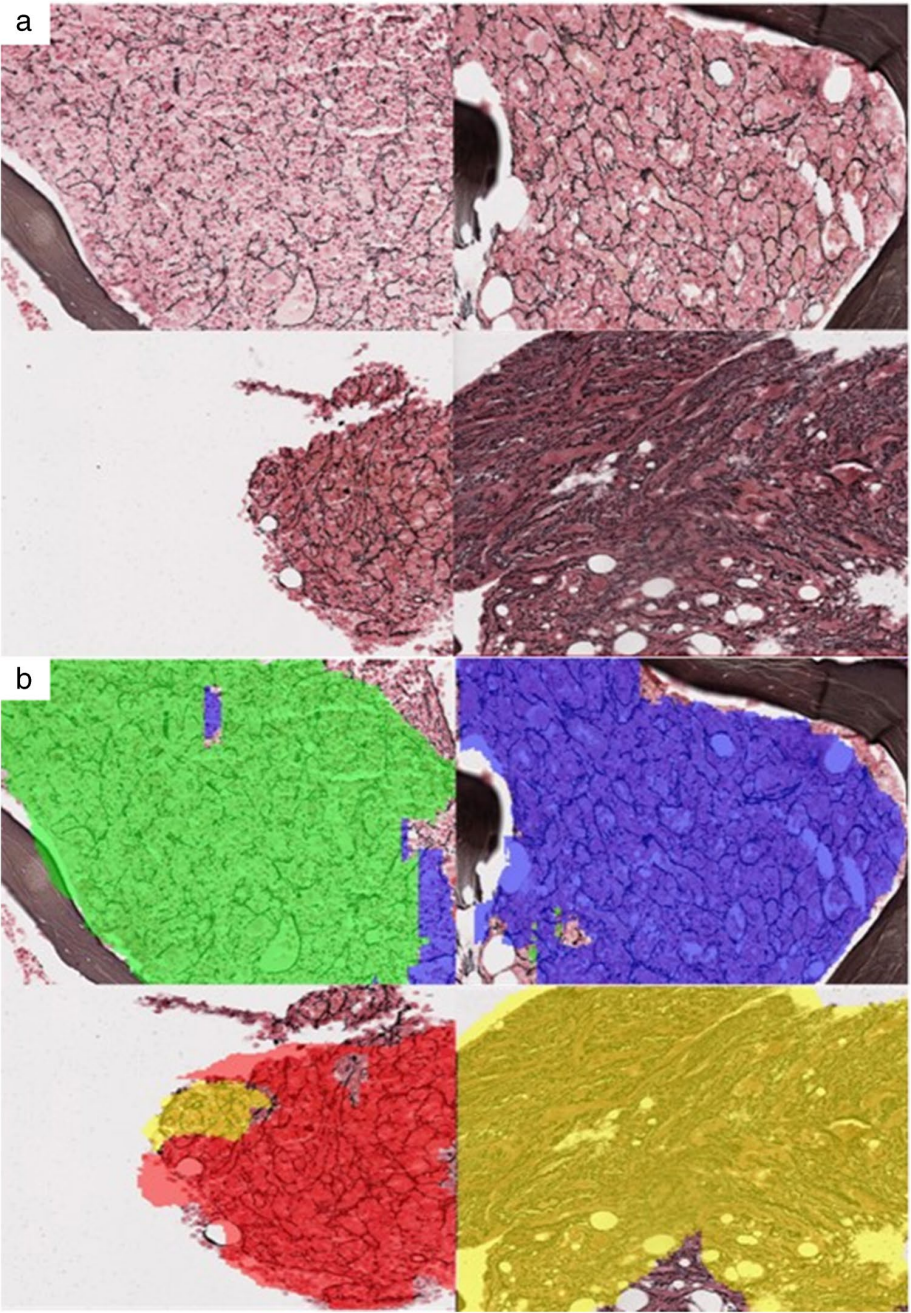
Example of evaluation of the model. **A** The patch selected for the evaluation (reticulin stain, 20ua. **B** Image provided

A specific QuPath script [18] was used to export the annotations, generating.tiff images (resolution 256 × 256, magnification 20 ×) and.png masks with four main colors (Fig. 1). In total, 3814 images and 3814 masks were obtained, and they composed our training set.

A semantic segmentation model for fibrosis assessment was developed using a fully convolutional network (FCN) based on the InceptionV3 architecture. The base architecture was extended with 9 custom convolutional and deconvolutional layers, culminating in a 1 × 1 convolution for pixel-by-pixel classification. Finally, the network was trained for 35 epochs using the 3814 images derived from the annotations.

### Validation and clinical application of the algorithm

To validate the developed algorithm, the remaining 40 BMBs were analyzed. A custom script was created using the FAST library for Python [19]. This script enabled tissue segmentation and fibrosis grading of the WSIs.

The WSIs were segmented into 256 × 256-pixel patches at 20 × magnification. The algorithm was applied to each patch to identify the specific grade of fibrosis, with labels extracted and overlaid on the tissue. The processed results were reconstructed into complete slides showing fibrosis segmentation.

**Fig. 2:**
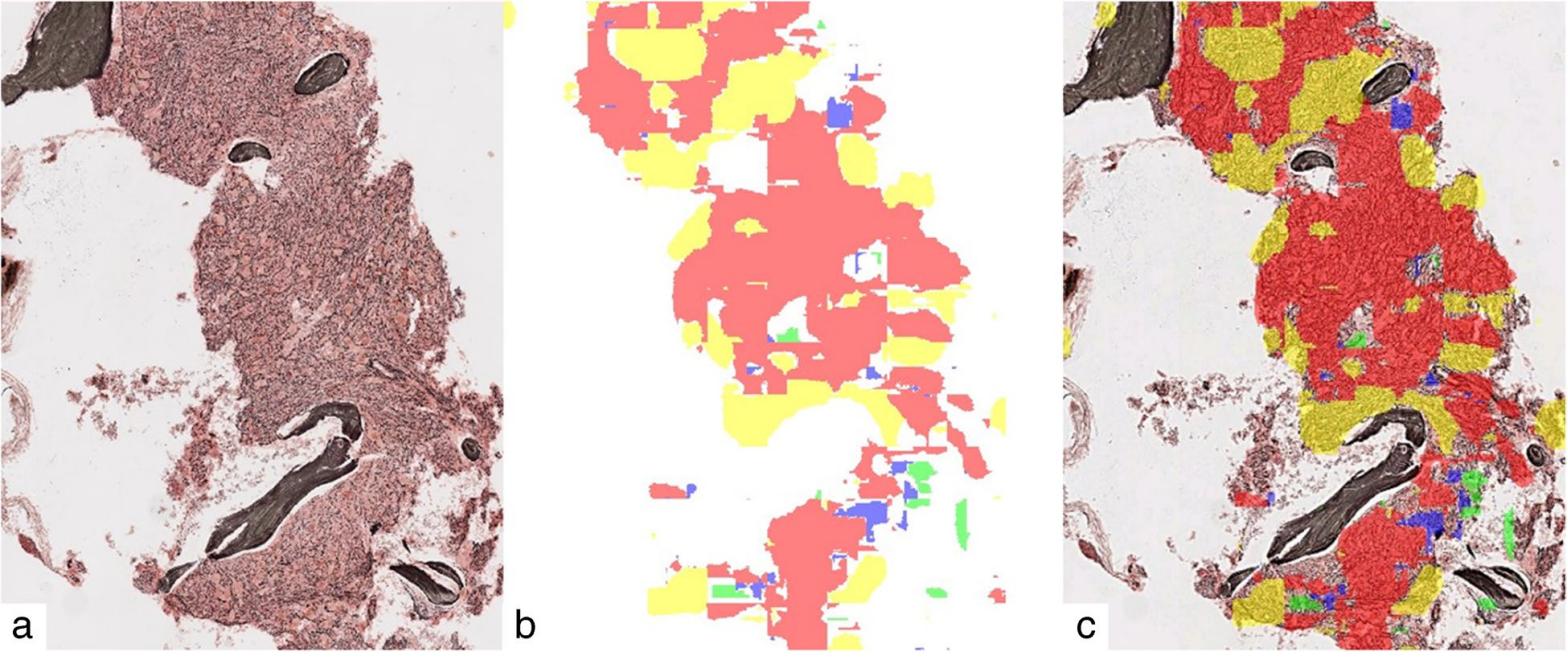
Workflow of segmentation process: WSI (**A**), segmentation result (**B**), WSI with overlaid segmentation image (**C**). Green, MF0; blue, MF1; red, MF2; yellow, MF3

Due to heterogeneity in the segmentation across entire WSIs, 40 representative patches (one per WSI) were selected for detailed evaluation based on the consistency of the algorithm’s assessments. These patches included both raw histological images and their segmented counterparts (Fig. 2).

An independent evaluation of fibrosis grades was conducted on the selected patches by a second expert hematopathologist (RF). The fibrosis scores assigned by the hematopathologist were compared with those generated by the algorithm. Concordance was quantified using Cohen’s kappa coefficient [20].

To assess the algorithm’s practical applicability in diverse clinical settings, including compatibility with different scanner technologies, a collaborative study was undertaken with pathology departments from three hospitals in the Campania region: Pagani Hospital, IRCCS Pascale, and Avellino Moscati Hospital. These institutions provided BMB specimens that met the inclusion criteria along with their corresponding histopathological reports.

A total of 21 BMBs were included in this phase, including 6 cases diagnosed as MF-0, 7 cases diagnosed as MF-1, 6 cases diagnosed as MF-2, and 2 cases diagnosed as MF-3. Reticulin-stained histological slides were prepared for each specimen and scanned using the Epredia P1000 scanner (Epredia, Portsmouth, NH, USA). The resulting WSIs were exported and analyzed using our algorithm.

### Quantitative reticulin fibrosis assessment and final grade determination

To provide a standardized fibrosis evaluation for the entire slide, a secondary script was developed to calculate the percentage distribution of each reticulin fibrosis grade in the segmented areas.

Following the World Health Organization (WHO) guidelines, the final fibrosis grade for each case was determined as the highest grade present in at least 30% of the hematopoietic tissue area [14].

Finally, reticulin fibrosis grades determined by the algorithm were compared with those provided by hematopathologists from the collaborating institutions. The level of agreement was evaluated using Cohen’s kappa coefficient to assess consistency between the automated and expert-derived assessments.

### Hardware and software

Annotations and classifications for the training dataset were performed using QuPath software version 4.3. Image data and corresponding masks were exported using a custom Groovy-based script.

The segmentation model was developed in Python 3.10.12 on the Google Colaboratory platform, leveraging a pro account to access additional computational resources, including extended RAM and NVIDIA V100 GPUs.

Scripts for model verification, calculation of fibrosis class percentages, and statistical analysis were also written in Python 3.10.12, utilizing the Anaconda® distribution. Development and execution were carried out in a Jupyter Notebook environment for streamlined programming and reproducibility.

All computational tasks were further executed on an Alienware Aurora® R11 workstation equipped with the following specifications: processor, Intel® Core™ i5-10600 KF CPU (4.10 GHz); memory, 64 GB of RAM; graphics, AMD Radeon™ RX 5700 XT GPU with 8 GB of dedicated memory.

This configuration ensured efficient processing of image data and facilitated the complex calculations required for model development and analysis.

## Results

**Fig. 3:**
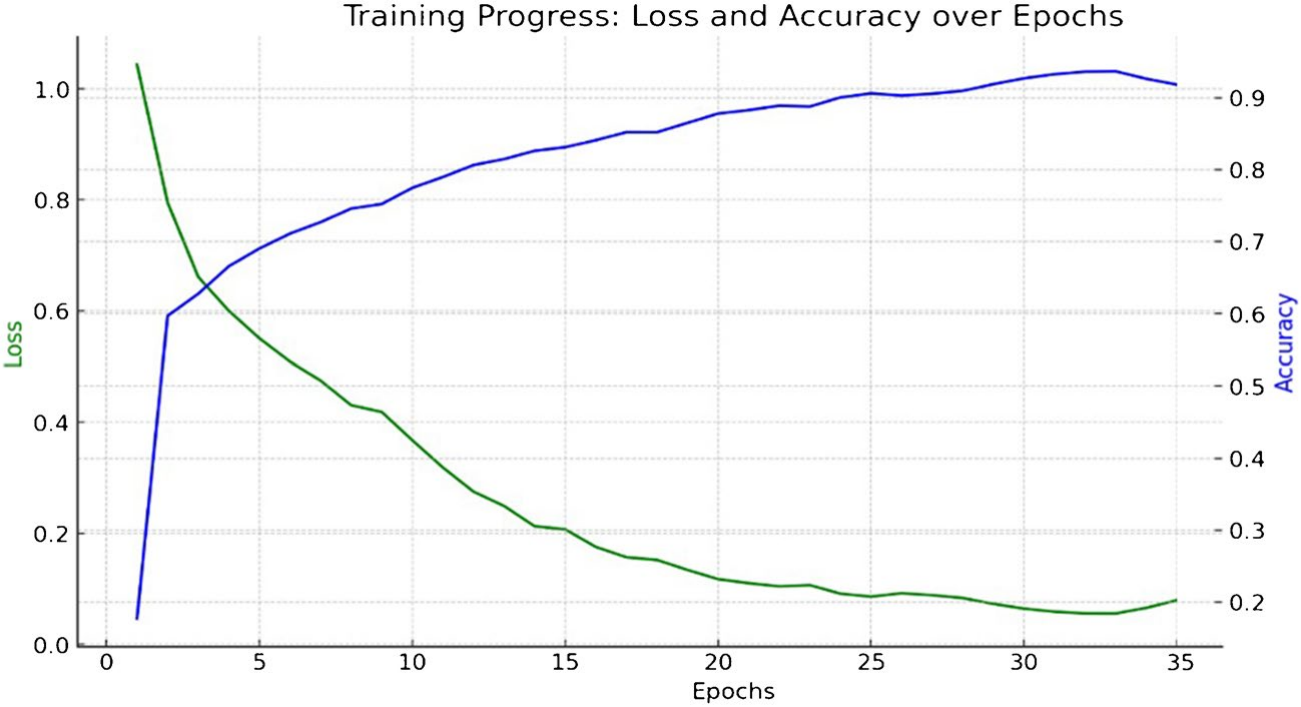
Graph of the training process: loss of function (green) and accuracy (blue)

The training process exhibited a positive trend, with rapid convergence of accuracy and loss function values toward optimal levels (accuracy > 0.9 and loss < 0.1) (Fig. 3).

The algorithm’s performance in assessing reticulin fibrosis was compared to evaluations conducted by an expert hematopathologist. This comparison yielded excellent overall agreement, as measured by Cohen’s kappa coefficient (*k* = 0.831). Agreement was particularly strong for specific fibrosis categories, with kappa values of 0.918 for MF0 and 0.886 for MF3, and substantial agreement for MF2 (*k* = 0.776) and MF1 (*k* = 0.749). All results were statistically significant (*p* < 0.001). A summary of these results is presented in Table 1.

**Table 1:** Agreement between model and pathologist in evaluation fibrosis on selected patches

	Cohen’s *K*	*p*-value	Level of agreement
General agreement	0.831	< 0.001	Optimal
MF-0	0.918	< 0.001	Optimal
MF-1	0.776	< 0.001	Substantial
MF-2	0.749	< 0.001	Substantial
MF-3	0.886	< 0.001	Optimal

Additionally, comparisons between the algorithm’s evaluations and those of hematopathologists from other hospitals showed consistency with the validation phase results. The overall agreement, measured using Cohen’s kappa coefficient, was excellent (*k* = 0.816). Agreement for specific fibrosis categories was optimal for MF0 (*k* = 0.893) and substantial for MF1 (*k* = 0.721) and MF2 (*k* = 0.775). As in the initial validation, all results were statistically significant (*p* < 0.001).

For MF3, the agreement was perfect (*k* = 1); however, this result lacked statistical significance due to the small sample size—only two BMBs were classified as MF3 in the corresponding reports, and both received identical evaluations from the algorithm.

A summary of these results is provided in Table 2.

**Table 2:** Agreement between model and fibrosis evaluation in BMBs provided by external institutions

	Cohen’s *K*	*p*-value	Level of agreement
General agreement	0.816	< 0.001	Optimal
MF-0	0.893	< 0.001	Optimal
MF-1	0.721	< 0.001	Substantial
MF-2	0.775	< 0.001	Substantial
MF-3	1	-	-

## Discussion

The histological evaluation of bone marrow fibrosis is a parameter of great importance both for defining the diagnosis of hematological bone marrow disorders and for determining the appropriate management of the patient. Such evaluation is based on the interpretation performed by the pathologist on histological sections stained using histochemical techniques for reticular fibres. However, it is a subjective and operator-dependent assessment, influenced by the specific pathologist’s experience. A digital tool capable of automatically assessing bone marrow fibrosis could help make the evaluation more uniform, standardized, and independent of the operator. Additionally, it could serve as a valuable training tool for pathologists in training and for residents, especially in a historical period when the number of pathologists is limited [21, 22].

The aim of this study was to develop a digital system for evaluating bone marrow reticulin fibrosis. In our study, we chose to scan the sections using high-resolution scanners (Ventana DP200 and Epredia P1000) approved for in vitro diagnostics to ensure precise quantitative and qualitative analysis. The annotations and classification of fibrosis were conducted by an expert hematopathologist, ensuring an ideal standard for training the algorithm [23].

Furthermore, we opted for a pre-trained neural network (Inception V3), which has shown promising results in training tools for histological evaluation [24].

We specifically developed a fully convolutional network (FCN) based on InceptionV3, leveraging transfer learning through the utilization of this architecture as the backbone. The network was adapted for the task of semantic segmentation by removing the fully connected classifier and incorporating a decoder composed of Conv2DTranspose layers to perform deconvolutional upsampling. The selected loss function was categorical crossentropy, which is well-suited for multi-class segmentation tasks, and it was combined with class weights to mitigate the effects of class imbalance, particularly addressing underrepresented classes, such as regions of advanced pathological tissue. Optimization was performed using the Adam algorithm, and the model was trained on a preprocessed dataset, where image normalization and the transformation of segmentation masks into one-hot encoded representations were applied.

The results of our study highlight the effectiveness of the developed algorithm. Specifically, both in the validation phase (where fibrosis was assessed on a single image) and in the comparison with pathologists from other institutions (where reticulin fibrosis was evaluated on the entire biopsy core scanned with a different scanner), the agreement between the model and expert pathologists was excellent (*K* > 0.8) for overall assessments and for the MF0 and MF3 categories. A substantial level of agreement (0.6 < *K* < 0.8) was observed for the MF1 and MF2 categories. These results suggest that the algorithm delivers fibrosis assessments on par with those of expert hematopathologists, who currently serve as the gold standard for this type of evaluation. In fact, Pozdnyakova et al. demonstrated that, using the WHO evaluation system, the agreement among expert pathologists in grading fibrosis in bone marrow biopsies (BMBs) is comparable to our findings (*K* = 0.84). However, it is not surprising that in both our study and Pozdnyakova’s, the concordance was slightly lower for intermediate grades (MF1 and MF2), as these categories are more susceptible to interpretative challenges [13].

Some studies have previously utilized image analysis or artificial intelligence to evaluate fibrosis in BMBs. Ryou et al. [25] employed a machine learning system to generate continuous maps of bone marrow fibrosis and to identify microfoci of advanced fibrosis, which are challenging to detect visually. The system achieved high accuracy, even when compared to expert hematopathologists. Teman et al. [26] developed a digital approach based on colour deconvolution to objectively quantify the area occupied by reticulin fibres, yielding numerical scores that showed a strong correlation with subjective grading systems (Bauermeister and European Consensus). The algorithm’s results were compared with those of three expert pathologists, demonstrating high accuracy and agreement, with excellent correlation and concordance coefficients (Spearman’s rho up to 0.78 and Cronbach’s alpha ≥ 0.90).

However, despite these studies achieve an accuracy comparable to our results, to the best of our knowledge, none of these studies tested the model on BMBs obtained from different institutions and/or digitized using different scanners. This aspect constitutes a key strength of our work, as it is well-established that the optical and computational properties WSIs can be affected by technical variations between scanners, as well as by differences in staining protocols across laboratories. Such factors may influence the performance of AI-based tools, highlighting the robustness of our algorithm in overcoming these potential challenges [27, 28].

Despite the promising results, this study has several limitations. The primary limitation is the model’s dependence on programming expertise (specifically Python) for implementation, which currently restricts its accessibility to pathologists with limited computational knowledge. Additionally, the small sample size used for external validation may limit the generalizability of the findings. In this study, the target of the research was the assessment of fibrosis based on reticular fibres and did not include assessment of collagen and osteosclerosis. Future efforts should focus on creating a user-friendly interface to facilitate the integration of the model tested in this study with others previously developed by our research group [16]. Furthermore, future studies should aim to validate the model on a larger sample size sourced from external institutions to enhance the robustness and generalizability of the results, and integrate the assessment of collagen and osteosclerosis into the system.

## Data Availability

The data that support the findings of this study are available from the corresponding author upon reasonable request.
